# Plant–*Agrobacterium* interaction mediated by ethylene and super-*Agrobacterium* conferring efficient gene transfer

**DOI:** 10.3389/fpls.2014.00681

**Published:** 2014-12-03

**Authors:** Satoko Nonaka, Hiroshi Ezura

**Affiliations:** Gene Research Center, Faculty of Life and Environmental Sciences, University of Tsukuba, Tsukuba, Japan

**Keywords:** *Agrobacterium*-mediated gene transfer, *Agrobacterium tumefaciens*, ACC deaminase, ethylene, *Agrobacterium*—plant interaction

## Abstract

*Agrobacterium tumefaciens* has a unique ability to transfer genes into plant genomes. This ability has been utilized for plant genetic engineering. However, the efficiency is not sufficient for all plant species. Several studies have shown that ethylene decreased the *Agrobacterium*-mediated transformation frequency. Thus, *A. tumefaciens* with an ability to suppress ethylene evolution would increase the efficiency of *Agrobacterium*-mediated transformation. Some studies showed that plant growth-promoting rhizobacteria (PGPR) can reduce ethylene levels in plants through 1-aminocyclopropane-1-carboxylic acid (ACC) deaminase, which cleaves the ethylene precursor ACC into α-ketobutyrate and ammonia, resulting in reduced ethylene production. The whole genome sequence data showed that *A. tumefaciens* does not possess an ACC deaminase gene in its genome. Therefore, providing ACC deaminase activity to the bacteria would improve gene transfer. As expected, *A. tumefaciens* with ACC deaminase activity, designated as super-*Agrobacterium*, could suppress ethylene evolution and increase the gene transfer efficiency in several plant species. In this review, we summarize plant–*Agrobacterium* interactions and their applications for improving *Agrobacterium*-mediated genetic engineering techniques via super-*Agrobacterium*.

## INTRODUCTION

*Agrobacterium tumefaciens* is a soil-borne bacterium known to cause crown gall disease in plants. *A. tumefaciens* strains that induce crown gall have a large plasmid (tumor-inducing plasmid: Ti plasmid), which is essential for the establishment of crown gall disease (van Larebeke et al., 1974, 1975; Zaenen et al., 1974; Watson et al., 1975; Currier and Nester, 1976). Chilton et al. (1977) detected part of the Ti plasmid in the crown gall genome, showing that plant cell conversion resulted in genetic transformation. This work clearly showed that *A. tumefaciens* has the ability to transfer T-DNA into plant genomes (Chilton et al., 1977).

Since this discovery in 1977, many investigators have attempted to generate *A. tumefaciens* suitable for plant genetic engineering. In early techniques, the oncogenic T-DNA region of the Ti plasmid was replaced with genes of interest by single or double homologous recombination (Zambryski et al., 1983). Although the recombination steps are easy, they are limited by the potential recombination of repetitive sequences during or after recombination in the Ti plasmid replicon in *A. tumefaciens*. Hoekema et al. (1983) showed that *A. tumefaciens* is able to deliver the T-DNA even if the Ti plasmid is divided into two plasmids (the T-DNA region and the *vir* region). This finding made it possible to extract only the T-DNA region from *A. tumefaciens*, and the modification was easier without the sequence limitation. After this finding, a binary plant vector strategy became the standard method in plant genetic engineering (Hoekema et al., 1983; Bevan, 1984). Increasing the virulence of *A. tumefaciens* is a useful strategy for higher transformation efficiency. The identification and application of *vir* gene inducers, such as plant phenolic compounds (Stachel et al., 1985, 1986) and sugars (Cangelosi et al., 1990; He et al., 2009; Hu et al., 2013), enabled this technique to be adapted for a wide range of plant species, especially monocot crops. The selection of hyper-virulent strains also spread the adaptation throughout the host range. Super-binary vectors, which have shown higher *vir* gene expression, have provided critical improvements in transformation efficiency (Komari et al., 2006). *Agrobacterium*-mediated gene transfer has been well established through many modifications, and it is a basic technique in plant science, but the gene transfer efficiencies are not sufficient for many plant species, especially crops and biomass plants. Therefore, improvements in these techniques are required.

Some plant metabolites and phytohormones inhibit *Agrobacterium*-mediated transformation. In particular, ethylene showed a negative effect on *Agrobacterium*-mediated T-DNA transformation in many plant species. In this review, we focus on the effect of ethylene on *Agrobacterium*-mediated gene transfer into plant cells and introduce an engineering strategy by which to increase the transformation efficiency of *A. tumefaciens* via ethylene removal. The established *A. tumefaciens* strain is designated as super-*Agrobacterium*.

## ETHYLENE SUPPRESSES *Agrobacterium*-MEDIATED T-DNA TRANSFER

The gaseous phytohormone ethylene regulates multiple physiological and developmental processes in plants, such as seedling emergence, leaf and flower senescence, ripening, organ abscission, growth-phase transitions, rhizobium–legume interaction, and plant–pathogen interaction (Beyer, 1981; Yang and Hoffman, 1984; Yang, 1985; Abeles, 1992; Ogawara et al., 2003). Ethylene synthesis is stimulated by biotic or abiotic stress. Ethylene also modulates *A. tumefaciens*–plant interactions. Ethylene is a crucial determinant of crown gall development. Plants treated with inhibitors of ethylene synthesis or perception, such as aminoethoxyvinylglycine (AVG), and ethylene-insensitive *Never-ripe* mutants (tomato) suppress crown gall growth (Aloni et al., 1998). Because vascularization is essential for efficient assimilate import from the host plant into the tumor cells, if the vascularization is suppressed, nutrient supply stops (Malsy et al., 1992; Pradel et al., 1996, 1999), resulting in the inhibition of crown gall development. Ethylene stimulates crown gall development by inducing vascular development (Wächter et al., 2003), and it induces crown gall development but inhibits *Agrobacterium*-mediated genetic transformation of plant cells. The enhancement of ethylene production by supplying its immediate precursor, 1-aminocyclopropane-1-carboxylic acid (ACC), suppresses gene transfer in tomatoes and melons (Davis et al., 1992; Ezura et al., 2000). Ethylene is increased by wounding and *A. tumefaciens* infection stress during co-cultivation (Ezura et al., 2000). Reducing ethylene production during co-cultivation using ethylene biosynthesis inhibitors such as AVG or suppressing plant ethylene perception by adding silver ions to the tissue culture medium has improved the transformation efficiency in melons (Ezura et al., 2000), cauliflowers (Chakrabarty et al., 2002), apricots (Burgos and Alburquerque, 2003), apple trees (Petri et al., 2005; Seong et al., 2005), and bottle gourds (Han et al., 2005). The stable transformation frequency was also increased in the *Arabidopsis thaliana* ethylene-insensitive mutants *etr1-1* and *etr1-2* (Nonaka et al., 2008a).

These results indicate that ethylene inhibits gene transfer in plants. One possible explanation for this phenomenon might involve the plant defense response via ethylene signaling. Previous studies have found that ethylene regulates several genes that are involved in the defense response, including those that encode the PR proteins chitinase, β-1, 3-glucanase, and PR1 (Deikman, 1997) in addition to phytoalexin synthetic enzymes (Ecker and Davis, 1987), defensins (Penninckx et al., 1996), and hydroxy-Pro-rich glycoproteins (Toppan et al., 1982). These compounds suppress bacterial growth because of their antibacterial activity. Indeed, in tomatoes, the reduced expression of* LeETR4*, which encodes a tomato ethylene receptor, resulted in increased sensitivity to ethylene, an enhanced hypersensitive response, and the reduced growth of *Xanthomonas campestris* pv. *vesicatoria* compared to wild-type (WT) plants (Ciardi et al., 2000). Ethylene insensitivity results in reduced resistance to the potato soft rot agent *Erwinia carotovora* subsp. *carotovora* in *A. thaliana etr1-1* and *ein2-1* mutants (Norman-Setterblad et al., 2000).

**FIGURE 1:**
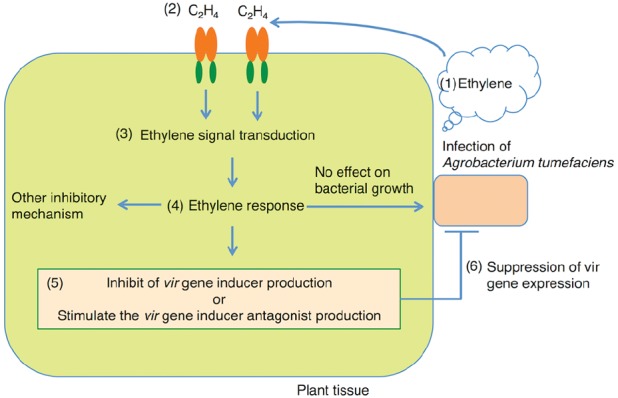
**Schematic drawing of the potential mechanisms of inhibition through ethylene signaling and *vir* gene expression. (1)** The infection of *A. tumefaciens* induces ethylene evolution from plant tissue. **(2)** Ethylene is received by the ethylene receptor. **(3)** Ethylene signal transduction is induced and, **(4)** subsequently, the plant shows the ethylene response. **(5)** In plants that show the ethylene response, *vir* gene inducer production would be suppressed or an antagonist of the inducer would be produced, **(6)** resulting in a reduction of *vir* gene expression in *A. tumefaciens.*

In general, ethylene levels increase in plant defense responses; however, the inhibitory effect of ethylene on *Agrobacterium*-mediated gene transfer does not occur through plant defense. The model scheme is described in Figure 1. Microarray and differential display analysis showed that *A. tumefaciens* infection induces the plant genes necessary for transformation while simultaneously repressing host defense response genes (Veena et al., 2003). Ethylene evolution was induced by *A. tumefaciens* inoculation in the early stage of the infection (Nonaka et al., 2008b; Lee et al., 2009). Although *A. tumefaciens* infection increased ethylene levels, the plant genes that encode ethylene receptors and their downstream signaling components, including defense response genes, are not induced (Lee et al., 2009). These results support the hypothesis that the inhibitory effect of ethylene on *Agrobacterium*-mediated gene transfer is independent of the plant defense response. Indeed, the plant ethylene response did not affect *A. tumefaciens* growth during co-cultivation (Nonaka et al., 2008a). The plant ethylene response inhibits T-DNA transfer through the suppression of *vir* gene expression (Nonaka et al., 2008a). The application of exudate from plants showing an ethylene response to *A. tumefaciens* reduced *vir* gene expression, which decreased T-DNA transfer. Such an inhibitory effect was partially overcome by the application of acetosyringone, a *vir* gene inducer, and in an *A. tumefaciens* strain constitutively expressing the *vir* gene. From this result, two possibilities were considered: in plants showing an ethylene response, the amount of the *vir* gene inducer would be reduced or the antagonist of the *vir* gene inducer would be produced. This deficient recovery indicates that *vir* gene suppression is one of the reasons for a reduction in T-DNA transfer via the ethylene response. The partial restoration upon the application of the *vir* gene inducer suggests that there is another inhibitory mechanism of ethylene. Therefore, the suppression of the *vir* gene is not sufficient to explain the negative effect of ethylene on *Agrobacterium*-mediated gene transfer. Because the inhibitory mechanism of ethylene on *Agrobacterium*-mediated gene transfer still needs to be clarified, to improve the transformation frequency, the introduction of the ability to reduce ethylene in *A. tumefaciens* would be effective.

## STRATEGY TO REDUCE ETHYLENE PRODUCTION IN PLANT CELLS INOCULATED WITH *Agrobacterium*

Ethylene is generated through the ethylene biosynthetic pathway, which was elucidated largely by the pioneering work of Yang and co-workers in the 1970s and 1980s (Wang et al., 2002). Ethylene is synthesized from the amino acid methionine, which is converted to *S*-adenosyl-*L*-methionine (SAM) by SAM synthase (ADS). SAM is the major methyl donor in plants and is involved in the methylation of lipids, proteins, and nucleic acids. SAM is converted by the enzyme ACC synthase (ACS) to 5′-methylthioadenosine (MTA), which is converted back to methionine via the Yang Cycle and to ACC, the precursor of ethylene. ACC is finally oxidized by ACC oxidase (ACO) to form ethylene, cyanide, and carbon dioxide. The conversion of SAM to ACC is considered to be the rate-limiting step in ethylene biosynthesis and consequently has been studied intensively. Therefore, the degradation of ACC, the immediate precursor of ethylene, effectively reduces ethylene production in plants. The reduction of ethylene via the degradation of ACC is found in some soil bacteria.

The pyridoxal 5-phosphate-dependent enzyme ACC deaminase catalyzes the decomposition of ACC to α-ketobutyrate and ammonia (Honma and Shimomura, 1978; Minami et al., 1998; Hontzeas et al., 2004; McDonnell et al., 2009). Although ACC deaminase genes have been isolated in a wide range of organisms, the ACC deaminase gene was isolated from many plant growth-promoting rhizobacteria (PGPR; Table 1). As shown in. PGPR with ACC deaminase activity can lower the ethylene level in plant cells through the degradation of ACC.

**Table 1:** Organisms with ACC deaminase activity.

**Source organisms**	**Reference**
Bacteria (Gram-negative)	
*Enterobacter cloacae*	Holguin and Glick (2001)
*Achromobacter xylosoxidans*	Belimov et al. (2001)
*Rhizobium leguminosarum*	Ma et al. (2003)
*Pseudomonas putida*	Hontzeas et al. (2004)
*Burkholderia phytofirmans*	Sessitsch et al. (2005)
*Variovorax paradoxus*	Madhaiyan et al. (2006)
*Methylobacterium fujisawaense*	Belimov et al. (2009)
*Cronobacter sakazakii*	Jha et al. (2012)
*Mesorhizobium* sp.	Gontia-Mishra et al. (2014)
*Haererehalobacter* sp.	Gontia-Mishra et al. (2014)
*Halomonas* sp.	Gontia-Mishra et al. (2014)
Bacteria (Gram-positive)	
*Rhodococcus* sp.	Belimov et al. (2001)
*Brevibacterium iodinum*	Dastager et al. (2010)
*Bacillus licheniformis*	Siddikee et al. (2011)
*Zhihengliuela alba*	Gontia et al. (2011)
*Micrococcus* sp.	Jha et al. (2012)
*Brachybacterium saurashtrense*	Gontia-Mishra et al. (2014)
*Brevibacterium casei*	Gontia-Mishra et al. (2014)
Archaebacteria	
*Pyrococcus horikoshii*	Fujino et al. (2004)
Yeasts	
*Hansenula saturnus*	Minami et al. (1998)
*Issatchenkia occidentalis*	Palmer et al. (2007)
Fungi	
*Penicillium citrinum*	Jia et al. (1999)
*Trichoderma asperellum*	Viterbo et al. (2010)
*Phytophthora sojae*	Singh and Kashyap (2012)
Plants	
*Arabidopsis thaliana*	McDonnell et al. (2009)
*Populus tremula*	Plett et al. (2009)
*Solanum lycopersicum*	Gontia-Mishra et al. (2014)

In response to tryptophan and other small molecules present in root exudates, IAA is synthesized and secreted by a PGPR that is bound to the surface of the root (Whipps, 1990; Hong et al., 1991; Fallik et al., 1994; Dimkpa et al., 2012). Some of the newly synthesized IAA is taken up by the plant and can stimulate ACS to convert SAM to ACC (Kende, 1993). The uptake and subsequent cleavage of ACC by the PGPR decrease the amount of ACC outside the plant (Penrose and Glick, 2001).

The *K*_m_ of ACC deaminase is lower than ACC oxidase. The various plant ACC oxidase for ACC ranges from approximately 8 μM (for ripening apples) to 120 μM (for etiolated beans; Smith et al., 1992). The *K*_m_ of ACC deaminase for ACC ranges from approximately 1.5 to 3.4 mM (Honma and Shimomura, 1978; Hontzeas et al., 2004). This means that ACC oxidase has about a 100-fold greater affinity for ACC than does ACC deaminase. Despite the fact that ACC oxidase has a much higher affinity for ACC, the kinetic calculations indicate that ACC deaminase can be more effective in lowering ACC than ACC oxidase when the amount of ACC deaminase is much greater than the amount of ACC oxidase (Glick et al., 1998). These results indicate that the ACC metabolite reaction in PGPR was more effective than that in plant cells and that the ACC level are lower in inner plant cells than in external cells. Therefore, to maintain equilibrium between internal and external ACC levels, the plant increases the level of ACC exudate. PGPRs have the ability to utilize ACC as a sole source of nitrogen in plant roots (Glick et al., 1994; Jacobson et al., 1994) and to proliferate under conditions in which other soil bacteria cannot grow. The reduction of the inner ACC level caused by the utilization of ACC in bacteria causes the plant to synthesize more ACC and to effectively exude ACC from the plant.

A significant correlation was found between *in vitro* bacterial ACC deaminase activity and the growth-promoting activity of these bacteria under pot and field trial conditions (Shaharoona et al., 2006a,b). In fact, many types of PGPR containing the ACC deaminase gene reduced ethylene production, resulting in physiological changes in many types of plants. The rhizobacterium *Variovorax paradoxus* 5C-2, which possesses ACC deaminase, promotes the growth (leaf area and shoot biomass) and development of WT *A. thaliana* and the ethylene-overproducing mutant *eto1-1* but does not have these effects in ethylene-insensitive mutants (*etr1-1* and *ein2-1*; Chen et al., 2013). Furthermore, *V. paradoxus* 5C-2 decreased the ACC concentrations in the rosette leaves of WT plants by 59% and foliar ethylene emission in both WT plants and *eto1-1* mutants by 42 and 37%, respectively, (Chen et al., 2013). Rhizobacteria possessing ACC deaminase induced maximum waterlogging tolerance in *Ocimum sanctum*, as treated waterlogged plants exhibited the maximum growth and biomass yield with minimal ethylene levels (Barnawal et al., 2012). Bacterial endophytes expressing ACC deaminase delay flower senescence (Ali et al., 2012). *Mesorhizobium ciceri* LMS-1 expressing an exogenous ACC deaminase increases nodulation abilities and chickpea plant resistance to soil constraints (Nascimento et al., 2012).

## SUPER-*Agrobacterium* WITH ACC DEAMINASE ACTIVITY INCREASES T-DNA TRANSFER EFFICIENCY VIA *A. tumefaciens*

**FIGURE 2:**
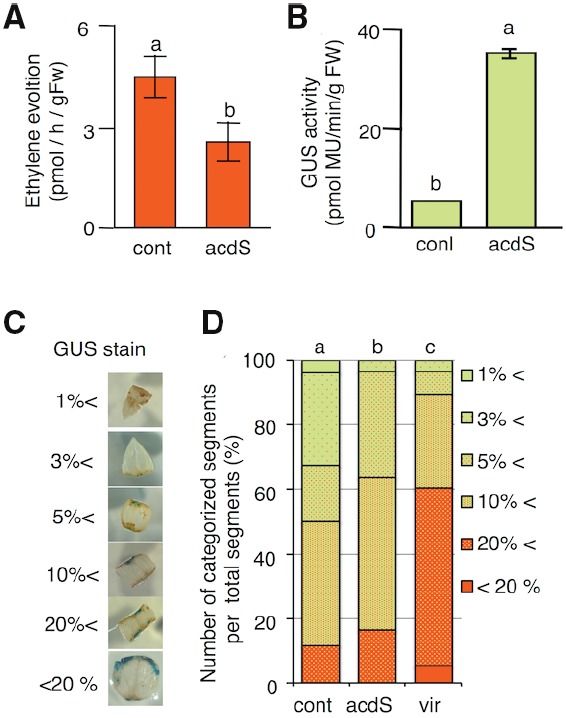
***Agrobacterium tumefaciens* with ACC deaminase activity inhibits ethylene evolution during co-cultivation, increasing gene transformation frequency in tomatoes and melons. (A)** Measurement of ethylene evolution. The accumulation of ethylene in the headspace was measured on a gas chromatograph. Bars represent standard deviations (*n* = 3). The characters a and b show significant differences (*t*-test; *P* < 0.05). fw, fresh weight. **(B)** Quantification of gene transfer by a GUS assay. Melon cotyledon segments were co-cultured with three different *A. tumefaciens* strains for 3 days. **(C)** Classification of GUS-stained cotyledon explants. GUS-stained tomato cotyledons were categorized based on the stained area: less than 1, 1–3, 3–5, 5–10, 10–20, and more than 20%. **(D)** The frequency of each GUS staining category in tomato explants. Bacterial strains with significant differences (Student’s *t*-test and Kruskal–Wallis test; *P* < 0.01) are indicated by different letters. cont: *A. tumefaciens* GV2260 (pBBR1MCS-5, pIG121); acdS: *A. tumefacien*s GV2260 (pBBR*acdS*, pIG121-Hm) *acdS* gene driven by lac promoter; virD1acdS: *A. tumefaciens* GV2260 (p*virD1acdS*, pIG121-Hm) *acdS* gene driven by the *virD1* promoter. Panels **(A,B)** are cited from Nonaka et al. (2008b). Panels **(C,D)** are cited from Someya et al. (2013).

The introduction of ACC deaminase activity into *A. tumefaciens* may reduce ethylene levels during co-cultivation and increase *A. tumefaciens*-mediated transformation efficiency. Whole-genome sequence analysis revealed that the *A. tumefaciens* strain C58 does not have an ACC deaminase gene (Wood et al., 2001); therefore, this strategy seems to be effective in this species. The ACC deaminase gene was amplified from *Pseudomonas* sp. via PCR. This amplified fragment was inserted into pBBR1MCS-5 (pBBR*acdS*). The pBBR1MCS-5 plasmid is compatible with IncP, IncQ, and IncW plasmids, and it has a different origin of the RK2 vector, which is used as a binary vector (Kovach et al., 1994). Because their origins are different, pBBR*acdS* and the binary vector are able to coexist in *A. tumefaciens*. The expression of ACC deaminase was controlled by the *lacZ* promoter, which constitutively and strongly expresses genes in *A. tumefaciens*. The *A. tumefaciens* strain harboring ACC deaminase genes showed ACC deaminase activity and effectively inhibited ethylene production in melons (Figure 2A), resulting in increased transient T-DNA transformation in melons (Figure 2B; Nonaka et al., 2008b).

For the further improvement of *Agrobacterium*-mediated transformation, we attempted to increase the expression level of ACC deaminase. Klüsener et al. (2010) showed that *virB1*, *virD1*, and *virE1* of *A. tumefaciens* were among the most highly expressed genes in acidic medium (AB medium at pH 5.5) containing 100 mol/L acetosyringone (Klüsener et al., 2010). We compared the activities of these three promoters to select the highest one, and the *virD1* promoter showed the strongest transcription activity. The *virD1* promoter conferred fourfold increased transcriptional activity compared with the *lacZ* promoter. The time course analysis (0–72 h) showed that ACC deaminase expression was induced at 6 h by adding acetosyringone to MS medium, and the high expression level was maintained until 72 h. By contrast, the expression of the ACC deaminase gene driven by the *lacZ* promoter started to decrease at 6 h. Compared with the *lacZ* promoter, the *virD1* promoter maintained ACC deaminase gene expression at a higher level for a long time, increasing the ACC deaminase activity in *A. tumefaciens*. Generally, the co-cultivation period is 72 to 96 h, so the *virD1* promoter seems to be suitable as the ACC deaminase gene activator. This newly developed *A. tumefaciens* strain showed higher T-DNA transformation efficiency in tomatoes (Figures 2C,D) and higher biomass production than *Erianthus ravennae*, which shows very low transformation frequency. Therefore, *A. tumefaciens* with higher ACC deaminase activity is a powerful tool for the *Agrobacterium*-mediated genetic engineering of plants (Someya et al., 2013). Introducing ACC deaminase into *A. tumefaciens* is effective at increasing the stable transformation frequency. *A. tumefaciens* with ACC deaminase succeeded in increasing the stable transformation frequency in Egusi melons (Ntui et al., 2009) and three canola cultivars (*Brassica napus* cv. Westar, *B. napus* cv. Hyola 401 and *B. napus* cv. 4414RR; Hao et al., 2010). We herein designate *A. tumefaciens* with ACC deaminase activity as super-*Agrobacterium*, which has the potential to improve the transformation efficiency of recalcitrant plant species.

## CONCLUSION

*Agrobacterium*-mediated transformation is an important tool for plant genetic engineering. Although a sophisticated protocol has been established for model plants, such as *A. thaliana*, tobacco, and rice, the transformation efficiency was not sufficiently high for commercially important crops such as maize, sorghum, soybean, barley, and *E. ravennae*. There has therefore been a need for the improvement of this methodology. Among the various negative factors contributing to low *Agrobacterium*-mediated transformation rates, ethylene has been well known as an inhibitor of transformation efficiency for a long time. There are chemicals to reduce ethylene production or ethylene perception. However, they are hard to use because they are expensive, in a gaseous form or toxic to bacteria; they are thus not suitable for improving *Agrobacterium*-mediated transformation. Therefore, we attempted to reduce ethylene in *A. tumefaciens*. Some PGPRs have an enzyme that degrades the ethylene precursor ACC. This strategy is not toxic for bacteria, and it is very easy to perform. We introduced the enzyme ACC deaminase into *A. tumefaciens.* The strain has been designated as super-*Agrobacterium*. It showed increased transient gene delivery into melon cotyledons (Nonaka et al., 2008b), tomatoes, and *E. ravennae* (Someya et al., 2013). In addition, the super-*Agrobacterium* also increased stable transformation in Egusi melon and three canola species (Ntui et al., 2009; Hao et al., 2010). To improve the “super-*Agrobacterium*,” a thorough analysis will be required in the future. One of the negative effects of ethylene is the suppression of *vir* gene expression in *A. tumefaciens*. However, the constitutive *vir* gene expression strain or the addition of the *vir* gene expression inducer could only partially overcome the inhibitory effect of ethylene on *vir* gene expression. This result showed the possibility of a different inhibitory mechanism that is caused by ethylene; therefore, a thorough analysis will be required in the future.

Our study showed that there are two points that should be considered when improving the efficiency of *Agrobacterium*-mediated gene transfer: first, the transient transformation frequency is important for increasing the stable transformation efficiency. Second, the regulation of plant-derived signals is an effective method for increasing the *A. tumefaciens*-mediated gene transfer frequency. For a more sophisticated strategy by which to increase transformation, it is effective to analyze the plant-derived signals in *Agrobacterium*–plant interactions. The removal of these signals would further increase the *A. tumefaciens*-mediated transformation frequency in recalcitrant plants.

### Conflict of Interest Statement

The authors declare that the research was conducted in the absence of any commercial or financial relationships that could be construed as a potential conflict of interest.
